# Identification of SORCS1 as a candidate gene associated with canine behavioral traits: Insights from guide dog training outcomes

**DOI:** 10.1371/journal.pone.0342346

**Published:** 2026-02-17

**Authors:** Toshinori Omi, Chihiro Udagawa, Yuiko Kato, Shota Kawakami, Yumiko Uno, Kazuhiko Ochiai, Junzo Asano

**Affiliations:** 1 Department of Basic Science, Nippon Veterinary and Life Science University, Tokyo, Japan; 2 Research Center for Animal Life Science, Nippon Veterinary and Life Science University, Tokyo, Japan; 3 Department of Chemistry, University of York, York, United Kingdom; 4 Department of Veterinary Hygiene, Nippon Veterinary and Life Science University, Tokyo, Japan; 5 Kyushu Guide Dog Association, Fukuoka, Japan; Chattogram Veterinary and Animal Sciences University, BANGLADESH

## Abstract

Although genetic factors contribute to behavioral variation in working dogs, the underlying molecular determinants remain poorly understood. The *sortilin-related VPS10 domain–containing receptor 1* (*SORCS1*) gene is highly expressed in the central nervous system and has been implicated in neuronal signaling and synaptic regulation. A preliminary genome-wide association study suggested an association between *SORCS1* variants and guide dog training outcomes. However, this result relied on only 28 dogs and lacked sufficient statistical power. In this study, we expanded the sample size and evaluated the association between *SORCS1* polymorphisms and behavioral suitability in 160 dogs, using pass/fail training outcomes as behavioral trait indicators. We initially validated 12 candidate SNPs within *SORCS1* using Sanger sequencing in 64 dogs, revealing significant genotype-dependent differences for eight loci located in predicted intron 3. A tagging SNP (rs23402730, C > T) was subsequently genotyped in 160 dogs (65 successful and 95 unsuccessful). The strongest association was detected under a recessive model (CC + CT vs. TT), yielding P = 4.7 × 10 ⁻ ⁵ and an odds ratio (OR) of 4.11 (95% CI: 2.07–8.18). As allele frequencies and genetic relatedness varied among dogs, we further evaluated the association using generalized linear mixed-effects models adjusting for sex, breed, and family structure. In the full dataset (n = 159), the additive genotype remained statistically significant (β = 2.20, P = 4.5 × 10 ⁻ ^4^; OR = 8.98, 95% CI: 2.64–30.59). Moreover, we confirmed the association in a genetically homogeneous subset of 114 Labrador retrievers, independent of breed effects (P = 0.029; OR = 4.71, 95% CI: 1.17–18.99). These results suggest that variation in *SORCS1* is associated with behavioral suitability in dogs, based on an expanded cohort with guide dog training outcomes. As behavior is polygenic, *SORCS1* represents one contributing locus that may be informative for future genetic studies aimed at elucidating the molecular basis of canine behavior.

## Introduction

Genetic diversity and molecular markers are widely used to characterize dog populations and detect gene polymorphisms underlying variation in physiological and behavioral traits [1–6]. Several genes have been implicated in behavioral regulation in dogs, particularly those involved in dopaminergic, serotonergic, and neuropeptide pathways that influence impulsivity, sociability, and stress responses. For example, genes involved in neurotransmitter pathways, such as the dopamine receptors (*DRD1*, *DRD4*) [6–9], tyrosine hydroxylase (*TH*) [9,10], dopamine transporter (*DAT/SLC6A3*) [11], glutamate transporter (*SLC1A2*) [12], and serotonin transporter (*SLC6A4/5-HTT*) [13,14], have been linked to behavior. Other associated genes include catechol-O-methyltransferase (*COMT*) [15,16], serotonin receptor subtypes (*HTR1D*, *HTR2C*, *5HT1A*, *5HT1B*, and *5HT1E2A*) [14,15], androgen receptor (*AR*) [17], oxytocin receptor (*OXTR*) [18–22], monoamine oxidase B (*MAOB*) [23], and stathmin 1 (*STMN1*) [24].

In particular, polymorphism in the tyrosine hydroxylase (*TH*) gene is associated with activity-impulsivity in German Shepherd Dogs [10], while *OXTR* variation has been linked to affiliative and human-directed social behavior in dogs [18]. These examples illustrate that genetic variation in neural signaling pathways could influence behavioral traits.

The sortilin-related Vps10p-domain–containing receptor 1 (SORCS1) is a biologically plausible candidate gene for behavioral regulation. SORCS1 is highly expressed in the central nervous system and plays critical roles in intracellular sorting and trafficking of synaptic receptors, including AMPA receptor complexes [25–27]. In addition, members of the Vps10p-domain receptor family have been implicated in synaptic plasticity and neurodegenerative disorders [28–33], and genomic analyses in foxes have identified *SORCS1* as a candidate gene associated with tame behavior [34]. These observations provide a biological rationale for examining *SORCS1* in relation to behavioral traits in dogs.

Guide dog training programs face persistent challenges worldwide due to low behavioral suitability and limited training success rates. Guide dog training outcomes reflect behavioral suitability because they encompass multiple cognitive and emotional dimensions, including learning aptitude, task persistence, stress tolerance, and social responsiveness in real operational environments [35–38]. Previous studies have applied training outcomes as objective behavioral indicators in working dogs as they capture stable behavioral traits relevant to professional performance [39–41]. Identifying genetic markers that predict success would improve selection efficiency and mitigate guide dog shortages [15,38,39].

Our preliminary genome-wide association study suggested that *SORCS1* may influence behavioral suitability in guide dogs. However, as the preliminary analysis was based on only 28 dogs, the sample size was extremely small, making the determination of whether the observed association represented a true biological signal or a false positive difficult. To clarify this uncertainty, the present study increases the sample size and conducts a focused candidate-gene analysis to evaluate the association of *SORCS1* variants with behavioral traits relevant to guide dog outcomes.

## Methods

### Dogs

A total of 160 blood samples provided by the Kyushu Guide Dog Association (Itoshima, Fukuoka, Japan) were used in this study (S1 Table, DNA Panels 1 + 2 + 3). The successful group consisted of 65 dogs, including 12 active guide dogs, 17 retired guide dogs, and 36 dogs that had successfully completed guide dog training. Conversely, the unsuccessful group comprised 95 dogs that failed guide dog training. Dogs that withdrew from training for reasons unrelated to behavioral suitability (e.g., illness or injury) were excluded from the unsuccessful group. The assessment criteria for guide dog suitability, as employed by the Kyushu Guide Dog Association, encompass a wide range of behavioral characteristics, including aggression, sensitivity, excitability, willingness, self-interest, suspicion, anxiety, adaptability, distractibility, dominance, and maturity. Such multidimensional evaluations highlight the complexity of behavioral traits underlying guide dog performance. Classification criteria for “successful” and “unsuccessful” training outcomes follow operational performance standards used by certified guide dog instructors and are generally based on procedures described in the Guide Dog Instructor Training Textbook published by the Japan Guide Dog Facilities Association [42]. In addition to the guide dog samples, genomic DNA samples from 220 dogs were used to examine single nucleotide polymorphism (SNP) distributions across different breeds. These samples were collected from six breeds commonly kept in Japan: Labrador Retrievers (n = 50), Golden Retrievers (n = 33), Chihuahuas (n = 35), French Bulldogs (n = 36), Toy Poodles (n = 36), and Shiba Inus (n = 30). All samples were collected, extracted, and stored in our laboratory as part of ongoing genetic studies.

Genomic DNA was extracted from peripheral blood using the QIAamp DNA Blood Mini Kit (Qiagen, Hilden, Germany). DNA concentration and purity were assessed using a NanoDrop 2000 spectrophotometer (Thermo Fisher Scientific, Waltham, MA, USA). All sample collection procedures were performed by licensed veterinarians from the Kyushu Guide Dog Association or the Veterinary Medical Teaching Hospital, Nippon Veterinary and Life Science University (NVLU). Written informed consent was obtained from all dog owners prior to sample collection.

### Validation of 12 SNPs in the canine *SORCS1* gene by Sanger sequencing

Twelve SNPs showing the strongest association signals within the *SORCS1* gene on chromosome 28 (S1 Fig) were selected based on a preliminary GWAS of 28 dogs (14 successful and 14 unsuccessful) performed using the CanineHD BeadChip 170K SNP array (Illumina, San Diego, CA, USA). The genomic positions of these SNPs were initially annotated based on the CanFam2.0 assembly. To reflect updates in genome annotation, the positions were re-evaluated and are shown according to the more recent CanFam3.1 assembly in Table 1. Corresponding rsIDs were identified using the Genome Data Viewer (Canis lupus familiaris, CanFam3.1), and these data are summarized in Table 1. For four SNPs, discrepancies in the designation of major and minor alleles were observed between the SNP array annotation and dbSNP records; in the present study, alleles were reported based on the rsID information. To validate these candidate SNPs within the *SORCS1* gene, all 12 loci were genotyped by Sanger sequencing of PCR-amplified products. Genomic DNA was obtained from 32 successful and 32 unsuccessful guide dogs (S1 Table, DNA Panels 1 + 2). Specific regions encompassing each SNP within *SORCS1* were amplified by PCR using primer sets designed for each locus (S2 Table). For consistency, subsequent references to SNPs use rs numbers.

**Table 1:** Selected SNP markers for the association study of the canine *SORCS1* gene and guide dog training outcomes.

Chr28 CanFam 2.0	Chr28 CanFam 3.1	Predected position**	Canine HD 170K SNP array	Major allele	Minor allele	rsID (dbSNP)	Major allele	Minor allele
21936584	18934555	5’ flanking region	BICF2G630271482*	T	C	rs23434110	C	T
21855809	18853780	intron	BICF2G630271608	G	A	rs23432775	G	A
21837933	18835904	intron	BICF2G630271618	T	C	rs23432735	T	C
21825320	18823291	intron	BICF2P677609	A	G	rs23419703	A	G
21822941	18820912	intron	BICF2P704802	C	A	rs23433778	C	A
21811200	18809171	intron	BICF2G630271644	G	A	rs23402767	G	A
21803517	18801488	intron	BICF2G630271656*	T	C	rs23402730	C	T
21783677	18781648	intron	BICF2G630271668	G	T	rs23402707	G	T
21754663	18752634	intron	BICF2G630271723	A	G	rs23418018	A	G
21712881	18710852	intron	BICF2G630271763*	A	G	rs23417942	G	A
21699367	18697338	intron	BICF2G630271774	A	G	rs23417911	A	G
21618399	18616370	3’ flanking region	BICF2S23061281	G	T	rs23415114	G	T

*SNP of the minor and major alleles are different between Canine HD 170K SNP array ID and rsID.

**NCBI Reference Sequence: NC_006610.3 and XM_005637877.2.

PCR reactions were performed in a 25 μL volume using FastStart *Taq* DNA Polymerase (Roche, Mannheim, Germany) according to the manufacturer’s instructions. Thermal cycling was conducted with a Veriti 96-well Thermal Cycler (Applied Biosystems, Foster City, CA, USA). Amplification products were confirmed by electrophoresis on 2% agarose gels stained with ethidium bromide (Nippon Gene Co., Ltd., Toyama, Japan).

PCR products were purified using the High Pure PCR Product Purification Kit (Roche, Mannheim, Germany) and subjected to direct sequencing using the BigDye Terminator v3.1 Cycle Sequencing Kit (Applied Biosystems). Sequencing reactions were purified with the BigDye XTerminator Purification Kit (Applied Biosystems) and analyzed on an ABI 3730 Genetic Analyzer (Applied Biosystems).

### Validation of a tagging SNP in the canine *SORCS1* gene

Among the 12 SNPs identified within the canine *SORCS1* gene, rs23402730 (BICF2G630271656) showed one of the strongest association signals and was located within a region of high linkage disequilibrium with neighboring SNPs. Based on both its strong association signal and its representativeness within this LD block, rs23402730 was selected as a tagging SNP for subsequent analyses. Genotyping of this SNP was performed by Sanger sequencing of PCR-amplified products or by real-time PCR using a TaqMan probe specifically designed for this study (S2 Table). We collected additional genomic DNA samples from 33 successful and 63 unsuccessful guide dogs (S1 Table 1, DNA Panel 3). Finally, genotyping of the *SORCS1* SNP rs23402730 (C > T) locus was carried out using genomic DNA from 160 dogs (S1 Table, DNA Panels 1 + 2 + 3), consisting of 65 successful and 95 unsuccessful guide dogs.

### Sequencing of the predicted coding region of the canine *SORCS1* gene using genomic DNA

To investigate coding region variants potentially associated with guide dog suitability, the predicted 27 exons of the canine *SORCS1* gene were amplified from the genomic DNA of two dogs homozygous for the informative CC genotype at the *SORCS1* SNP rs23402730 locus. PCR primers (S2 Table) were designed based on the canine *SORCS1* gene sequence (NCBI Reference Sequence: NC_006610.3), located on chromosome 28 of the *Canis lupus familiaris* Boxer genome (CanFam3.1, whole-genome shotgun sequence).

### Expression analysis of the *SORCS1* gene in canine tissues

To investigate the expression of the *SORCS1* gene, reverse transcription PCR (RT-PCR) was performed using cDNA synthesized from various canine tissues. cDNA samples were obtained from Zyagen (San Diego, CA, USA) and BioChain Institute, Inc. (Newark, CA, USA). The canine *SORCS1* cDNA, corresponding to the predicted exons 3–5 of the *SORCS1* gene, was amplified by PCR. The canine glyceraldehyde-3-phosphate dehydrogenase (*GAPDH*) gene was used as an internal control [43]. The PCR products were electrophoresed on 2% agarose gels, stained with ethidium bromide, and visualized under ultraviolet illumination.

### Statistical analyses

Statistical significance was evaluated using Fisher’s exact test (two-tailed), with P < 0.05 considered statistically significant. Odds ratios (ORs) and 95% confidence intervals (CI) were calculated using 2 × 2 contingency tables. Linkage disequilibrium (LD) was assessed by calculating r² values. These analyses were performed using SNPAlyze (Dynacom, Mobara, Japan) and BellCurve for Excel v4.09 (Social Survey Research Information Co., Ltd., Tokyo, Japan).

To further adjust for sex, breed, and family effects, generalized linear mixed-effects models (GLMMs) with a binomial distribution and logit link were used to test the association between *SORCS1* genotype and training outcome. Genotype was coded additively, sex and breed were included as fixed effects, and family was included as a random intercept. Models were fitted using the lme4 package in R (version 4.5.2), and results are reported as ORs with 95% CI. A secondary analysis was conducted in Labrador retrievers only.

### Ethics statement

All experimental procedures were approved by the Experimental Animal Ethics Committee of the Nippon Veterinary and Life Science University.

## Results

### Association between *SORCS1* variants and guide dog training outcomes

A previous preliminary genome-wide association analysis conducted in 2015 using 28 dogs (14 successful and 14 unsuccessful) identified a suggestive association signal on chromosome 28, indicating *SORCS1* as a potential candidate gene associated with guide dog suitability (S1 Fig). Based on the preliminary GWAS results, we selected the top 12 SNPs in the canine *SORCS1* gene with the strongest association signals for further analysis to evaluate whether the observed associations reflect true biological signals or false positives. To validate these associations, additional samples were collected, and association analyses between these 12 SNPs within *SORCS1* and guide dog training outcomes were performed in a cohort of 64 dogs (32 successful and 32 unsuccessful). Significant differences in genotype frequencies between successful and unsuccessful guide dogs were observed for eight of the 12 SNPs—rs23432735, rs23419703, rs23433778, rs23402767, rs23402730, rs23402707, rs23418018, and rs23417942—located within the *SORCS1* gene in predicted intron 3 (Genome Data Viewer; Canis lupus familiaris, CanFam3.1), when comparing major homozygotes versus all other genotypes or minor homozygotes versus all other genotypes (Table 2).

**Table 2:** Genotyping data and association analysis of 12 SNPs in the canine *SORCS1* gene and guide dog training outcomes in a cohort of 64 dogs (32 successful and 32 unsuccessful guide dogs).

rsID#	rs23434110	rs23432775	rs23432735	rs23419703	rs23433778	rs23402767	rs23402730	rs23402707	rs23418018	rs23417942	rs23417911	rs23415114
Successful guide dog group (n = 32)												
Major allele homozygotes	11 (34.4%)	12 (37.5%)	13 (40.6%)	13 (40.6%)	11 (34.4%)	17 (53.1%)	5 (15.6%)	12 (37.5%)	8 (25.0%)	13 (40.6%)	11 (34.4%)	14 (43.8%)
Heterozygous	10 (31.3%)	12 (37.5%)	12 (37.5%)	15 (46.9%)	16 (50.0%)	12 (37.5%)	16 (50.0%)	14 (43.8%)	10 (31.3%)	10 (31.3%)	7 (21.9%)	12 (37.5%)
Minor allele homozygotes	11 (34.4%)	8 (25.0%)	7 (21.9%)	4 (12.5%)	5 (15.6%)	3 (9.38%)	11 (34.4%)	6 (18.8%)	14 (43.8%)	9 (28.1%)	14 (43.8%)	6 (18.8%)
Major allele frequency	0.500	0.562	0.594	0.641	0.594	0.719	0.406	0.594	0.406	0.563	0.453	0.625
Minor allele frequency	0.500	0.438	0.406	0.359	0.406	0.281	0.594	0.406	0.594	0.438	0.547	0.375
Unsuccessful guide dog group (n = 32)												
Major allele homozygotes	5 (15.6%)	24 (75.0%)	28 (87.5%)	28 (87.5%)	28 (87.5%)	28 (87.5%)	0 (0.00%)	21 (65.6%)	0 (0.00%)	25 (78.1%)	19 (59.4%)	24 (75.0%)
Heterozygous	10 (31.3%)	6 (18.8%)	4 (12.5%)	4 (12.5%)	4 (12.5%)	4 (12.5%)	4 (12.5%)	10 (31.3%)	6 (18.8%)	7 (21.9%)	8 (25.0%)	7 (21.9%)
Minor allele homozygotes	17 (53.1%)	2 (6.25%)	0 (0.00%)	0 (0.00%)	0 (0.00%)	0 (0.00%)	28 (87.5%)	1 (3.13%)	26 (81.2%)	0 (0.00%)	5 (15.6%)	1 (3.13%)
Major allele frequency	0.313	0.844	0.938	0.938	0.938	0.938	0.063	0.813	0.094	0.891	0.719	0.859
Minor allele frequency	0.688	0.156	0.063	0.063	0.063	0.063	0.938	0.188	0.906	0.109	0.281	0.141
*P*												
Major allele homozygotes vs others	0.148	0.082	1.86E-04	1.86E-04	2.48E-05	5.42E-03	0.053	0.045	4.75E-03	4.66E-03	0.079	0.021
Minor allele homozygotes vs others	0.207	0.005	0.011	0.113	0.053	0.238	2.48E-05	0.104	4.04E-03	2.04E-03	0.027	0.104

#https://may2021.archive.ensembl.org/Canis_lupus_familiaris/Info/Index.

n: number.

Pairwise linkage disequilibrium (LD) among these loci was evaluated by calculating D′ and *r*² values using genotype data from a cohort of 64 dogs.

**Fig 1:**
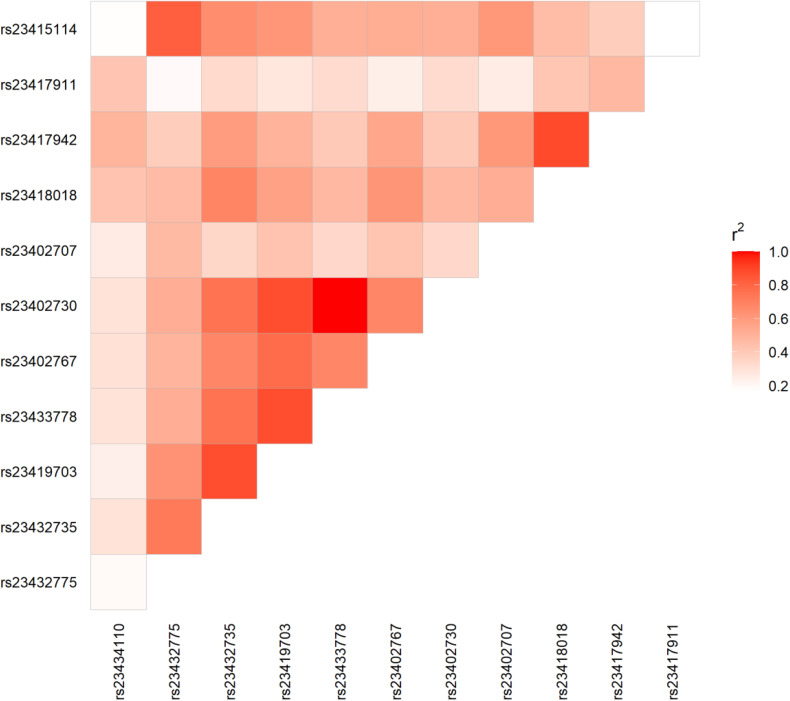
Linkage disequilibrium (LD) heat map based on pairwise r² values among SNPs. Color intensity indicates the strength of LD, ranging from low (white) to high (red). The heat map depicts pairwise LD among 12 SNPs within the canine *SORCS1* gene, evaluated in a cohort of 64 dogs comprising 32 successful and 32 unsuccessful guide dogs.

Strong LD was observed among rs23432735, rs23419703, rs23433778, rs23402767, and rs23402730, with D′ values ranging from 0.867 to 1.000 and *r*² values from 0.678 to 1.000 (S3 Table). These SNPs are located in predicted intron 3 of the canine *SORCS1* gene (NCBI Reference Sequences: NC_006610.3 and XM_005637877.2). Pairwise LD values (r²) are represented in an upper-triangular heat map shown in Fig 1.

Based on the results obtained from the 64-dog cohort, we further expanded the sample size to confirm the association in a larger dataset. To confirm the association between *SORCS1* genotype and guide dog training outcomes, we increased the sample size and genotyped dogs using a tagging SNP, rs23402730 (C > T), which showed one of the strongest statistical associations under a recessive model and was located within a region of high linkage disequilibrium with neighboring SNPs (Tables 2, S3 and Fig 1).

Genotype data for *SORCS1* SNP rs23402730 were obtained from a cohort of 160 dogs (65 successful and 95 unsuccessful in training). Table 3 summarizes the association between *SORCS1* SNP rs23402730 genotypes or alleles and guide dog training outcomes. The most significant difference was detected under a recessive genetic model (with the major allele considered recessive), when comparing CC + CT vs. TT genotypes (P = 0.0000474, OR = 4.11, 95% CI: 2.07–8.18).

**Table 3:** Association between the canine *SORCS1* SNP rs23402730 and guide dog training outcomes in a cohort of 160 dogs (65 successful and 95 unsuccessful guide dogs).

Genotype	Succesful Guide dog group (n = 65)	Unsuccessfule Guide dog group (n = 95)	*P*	Odds ratio	95% CI
CC	5 (7.69%)	1 (1.05%)			
CT	30 (46.2%)	20 (21.5%)			
TT	30 (46.2%)	74(77.9%)			
*Major allele dominant*			4.06E-02	7.83	0.893-68.7
CC	5(7.69%)	1(1.05%)			
CT + TT	60(92.3%)	94 (98.9%)			
*Major allele recessive*			4.74E-05	4.11	2.07-8.18
CC + CT	35 (53.8%)	21 (22.1%)			
TT	30 (46.2%)	74 (77.9%)			
*Allele frequency*			2.62E-05	3.39	1.9-6.06
C	0.308	0.116			
T	0.692	0.884			

n: number.

### Distribution of *SORCS1* SNP rs23402730 in different dog breeds

We examined the allele frequency of the canine *SORCS1* SNP rs23402730 in 220 dogs representing six breeds: Labrador Retrievers, Golden Retrievers, Chihuahuas, French Bulldogs, Toy Poodles, and Shiba Inus. Genotype and allele frequencies differed among breeds, with the frequency of the C allele ranging from 0.030 to 0.633 and 0.400 in total (S4 Table). These results indicate that *SORCS1* SNP rs23402730 is broadly distributed across diverse dog breeds, with differences observed among breeds.

### Association between *SORCS1* and guide dog suitability after adjusting for covariates

Because allele frequencies of *SORCS1* SNP rs23402730 differed considerably among breeds and several dogs in the training cohort were genetically related, we next evaluated the association between *SORCS1* and behavioral suitability using a mixed-effects model that accounted for potential confounding by breed composition, sex, and family structure. The initial dataset included 160 dogs; however, one Standard Poodle was excluded because it was the only dog of that breed. The final sample used for the full analysis consisted of 159 dogs. In the full dataset of 159 dogs, the additive *SORCS1* genotype was significantly associated with behavioral suitability after adjusting for sex, breed, and family effects (β = 2.20, SE = 0.63, P = 0.00045). The corresponding odds ratio was 8.98 (95% CI: 2.64–30.59), indicating that each additional allele increased the odds of successful guide dog qualification. Sex and breed did not show statistically significant contributions (Table 4). The variance component of the random effect (family) was substantial (SD = 1.62), indicating that clustering within family lines influenced behavioral outcomes and justified the use of a mixed model.

**Table 4:** Association between the canine *SORCS1* SNP rs23402730 and guide dog training outcomes in 159 dogs using generalized linear mixed-effects models.

Variable	β (SE)	Odds ratio (per allele)	95% CI	*P*
SNP (additive)	2.20 (0.63)	8.98	2.64–30.59	4.45E − 04
Sex (Male vs Female)	−0.18 (0.48)	–	–	0.71
Breed (Mixed vs LR)	−0.03 (0.67)	–	–	0.96

Sex and breed were included as fixed effects, and family, defined by the sire–dam pair, was included as a random intercept to account for pedigree-based relatedness.

To further evaluate robustness in a genetically homogeneous background, we repeated the analysis using only the 114 Labrador retrievers. Even in the absence of breed effects, the additive genotype remained statistically significant (β = 1.55, SE = 0.71, P = 0.029), with an odds ratio of 4.71 (95% CI: 1.17–18.99). Sex was not associated with training outcome in either model (Table 5).

**Table 5:** Within-breed association between the canine *SORCS1* SNP rs23402730 and guide dog training outcomes in 114 Labrador Retrievers using generalized linear mixed-effects models.

Variable	β (SE)	Odds ratio (per allele)	95% CI	*P*
SNP (additive)	1.55 (0.71)	4.71	1.17–18.99	0.029
Sex (Male vs Female)	−0.20 (0.57)	–	–	0.73

Family (random intercept).

This within-breed analysis was conducted to evaluate whether the observed association was independent of breed composition. Breed was not included as a covariate.

Taken together, these results indicated that the association between *SORCS1* genotype and guide dog suitability remains significant after adjustment for sex, breed, and family relatedness, and is also observed within a more genetically uniform subgroup.

### Preliminary screening for coding variation in *SORCS1*

As rs23402730 is located within predicted intron 3 of *SORCS1*, we conducted a preliminary screen of the predicted coding region to evaluate the potential contribution of exonic variants to functional differences. PCR amplification and sequencing of the coding regions were carried out in two dogs homozygous for the informative CC genotype. No functionally relevant coding variation was detected, suggesting that the observed association is unlikely to be explained by amino acid–altering mutations and may instead reflect regulatory effects.

### Expression of canine *SORCS1* gene in brain tissues

To determine whether the canine *SORCS1* gene is expressed in the brain, RT-PCR was performed using cDNA derived from various canine tissues. *SORCS1* cDNA was successfully amplified from several tissues, including the cerebral cortex, cerebellum, hypothalamus, hippocampus, and spinal cord (S2 Fig). These experiments were not designed to assess genotype-dependent expression differences; instead, they confirmed that the canine *SORCS1* gene is transcribed in brain tissues, which supports the biological plausibility of behavioral associations.

## Discussion

Behavioral suitability in guide dogs represents a complex, multifactorial phenotype. Although the present study analyzed 160 guide dogs, a modest sample size for genetic analyses of complex behavioral traits that may limit power to detect loci with small effects, the association between *SORCS1* SNP rs23402730 and training outcome showed a comparatively large effect size and remained statistically significant. In addition, the association between rs23402730 and guide dog training outcomes remained statistically significant after adjustment for breed, sex, and relatedness. While guide dog suitability is not determined by any single gene, the present findings suggest that *SORCS*1 provides biologically and empirically meaningful information as part of the polygenic basis of behavioral suitability in working dogs.

The biological plausibility of *SORCS1* as a behavioral candidate gene is supported by its high expression in the central nervous system and its established role in intracellular sorting and trafficking of synaptic receptors, including AMPA receptor complexes [25–27]. Previous studies have implicated *SORCS1* and related Vps10p-domain receptors in synaptic plasticity and neurodegenerative disorders [28–32], and recent genomic analyses in foxes identified *SORCS1* as a candidate gene associated with tame behavior [34]. Together, evidence from Alzheimer’s disease and fox domestication studies suggests that *SORCS1* influences behavioral and neurological phenotypes primarily through haplotypes composed of noncoding variants that affect gene expression, rather than through protein-altering coding mutations. In this study, we suggested *SORCS1* as a candidate gene associated with canine behavioral traits using *SORCS1* intronic SNPs. However, its functional consequences remain uncertain in this study. Therefore, the current data do not establish a mechanistic explanation for the association between *SORCS1* intronic SNPs and guide dog training outcomes. Future studies incorporating dense fine-mapping, haplotype analysis, allele-specific expression profiling, RNA-seq, and functional assays will be required to identify causal regulatory variants and to clarify how variation at the *SORCS1* locus influences neural or behavioral phenotypes in dogs.

## Limitations of the study

Clarifying the contribution of genetic diversity at the *SORCS1* locus to guide dog suitability will require analyses in larger and more diverse populations, as well as assessment of unmeasured environmental factors, including housing conditions, handler effects, and training practices. Future prospective studies with standardized environmental and management data will be essential to disentangle genetic and environmental influences.

## Conclusion

In this study, *SORCS1* was identified as a candidate gene associated with behavioral traits relevant to guide dog suitability. Given the multifactorial nature of canine behavior, these findings contribute to understanding the genetic components of behavioral variation and may provide insight into the genetic architecture of working dog performance. More broadly, the results underscore the importance of continued research to identify causal variants and mechanisms underlying animal behavioral traits.

## Supporting information

S1 Table Samples from a genetic association study between canine *SORCS1* SNPs and guide dog training outcomes. (XLSX)

S2 Table List of primers used for canine *SORCS1* gene analysis. (XLSX)

S3 Table Pairwise linkage disequilibrium (LD) measures of |D′| and r² among 12 SNPs of the canine *SORCS1* gene in a cohort of 64 dogs (32 successful and 32 unsuccessful guide dogs). (XLTX)

S4 Table Distribution of the canine *SORCS1* SNP rs23402730 among different dog breeds. (XLSX)

S1 Fig Manhattan plot from a preliminary genome-wide association study (GWAS) assessing for guide dog training out come in a small canine cohort. A suggestive association plot was detected on chromosome 28, indicating *SORCS1* as a potential candidate gene associated with guide dog suitability.
(PPTX)

S2 Fig RT-PCR analysis of canine *SORCS1* mRNA in various dog tissues. Dog *SORCS1* cDNA encompassing predicted exons 3–5 was amplified from different tissues. The integrity of RNA was confirmed using a primer pair for the *glyceraldehyde-3-phosphate dehydrogenase* (*GAPDH*) gene.
(PPTX)
