# Supplementary figures and images for: Identification of SORCS1 as a candidate gene associated with canine behavioral traits: Insights from guide dog training outcomes
Source: PLoS One. 2026 Feb 17;21(2):e0342346. doi: 10.1371/journal.pone.0342346 (PMC12912605; doi:10.1371/journal.pone.0342346)

## Slide 1
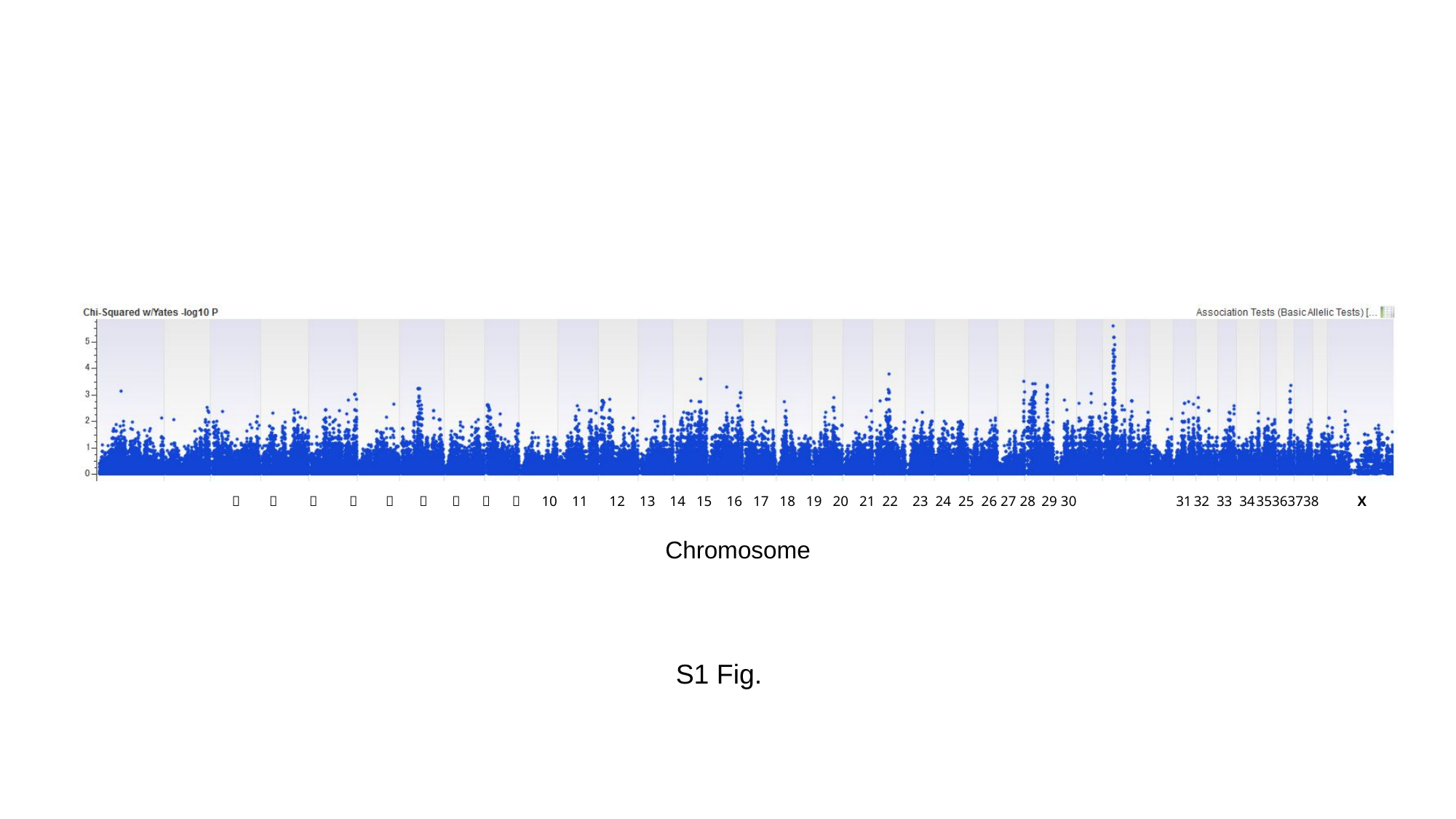

１ ２ 　 ３　 ４ 　５ ６　 ７ ８ ９ 10 11 12 13 14 15 16 17 18 19 20 21 22 23 24 25 26 27 28 29 30
31 32　33　34 35363738
X
Chromosome
S1 Fig.

Supplement: S1 Fig — A suggestive association plot was detected on chromosome 28, indicating SORCS1 as a potential candidate gene associated with guide dog suitability. (PPTX) [file pone.0342346.s005.pptx]
