# Supplementary material for: Identification of SORCS1 as a candidate gene associated with canine behavioral traits: Insights from guide dog training outcomes
Source: PLoS One. 2026 Feb 17;21(2):e0342346. doi: 10.1371/journal.pone.0342346 (PMC12912605; doi:10.1371/journal.pone.0342346)
Supplement: S2 Fig — Dog SORCS1 cDNA encompassing predicted exons 3–5 was amplified from different tissues. The integrity of RNA was confirmed using a primer pair for the glyceraldehyde-3-phosphate dehydrogenase (GAPDH) gene. (PPTX) [file pone.0342346.s006.pptx]

## Slide 1
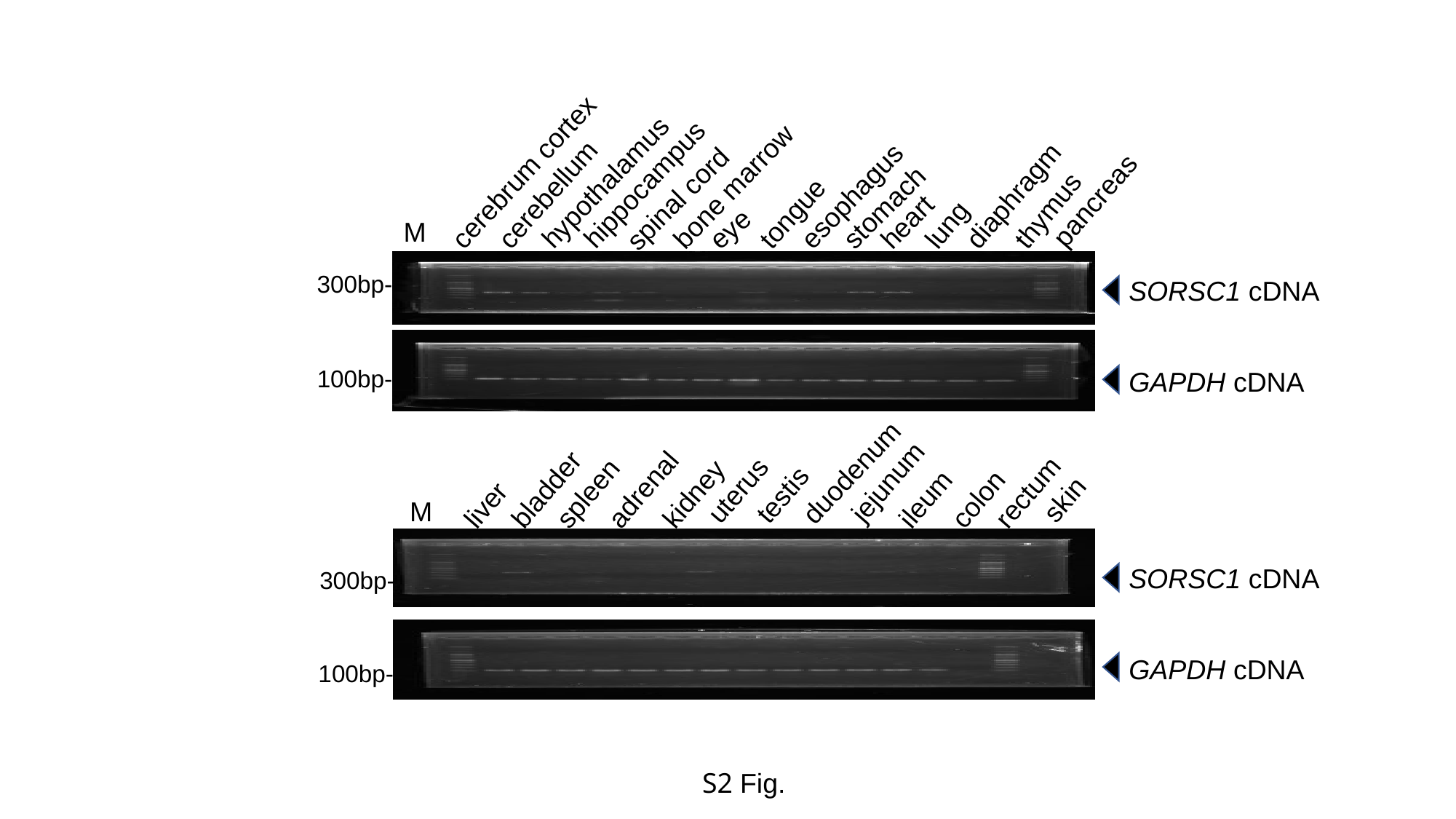

hypothalamus
cerebrum cortex
bone marrow
esophagus
hippocampus
cerebellum
spinal cord
 pancreas
diaphragm
thymus
tongue
lung
stomach
heart
eye
M
300bp-
SORSC1 cDNA
100bp-
GAPDH cDNA
duodenum
rectum
 jejunum
 uterus
liver
spleen
adrenal
kidney
bladder
 testis
ileum
colon
 skin
M
SORSC1 cDNA
300bp-
GAPDH cDNA
100bp-
 S2 Fig.
